# Serum Transthyretin Level as a Plausible Marker for Diagnosis of Child Acute Malnutrition

**DOI:** 10.1155/2017/9196538

**Published:** 2017-08-08

**Authors:** Behailu Tsegaye, Amha Mekasha, Solomon Genet

**Affiliations:** ^1^Biomedical Sciences Unit, School of Medicine, College of Health Sciences, Arba Minch University, Arba Minch, Ethiopia; ^2^Department of Pediatrics, School of Medicine, College of Health Sciences, Addis Ababa University, P.O. Box 9086, Addis Ababa, Ethiopia; ^3^Department of Medical Biochemistry, School of Medicine, College of Health Sciences, Addis Ababa University, P.O. Box 9086, Addis Ababa, Ethiopia

## Abstract

Malnutrition is a major underlying condition for mortality in children under five years of age in developing countries, particularly in Ethiopia. The most important forms of malnutrition in Ethiopia are protein and energy deficiencies. There is no reliable laboratory method at present to assess acute malnutrition. Transthyretin is a homotetrameric serum protein with half-life of two days. The main objective of this study was to assess the estimation of serum transthyretin level as a useful diagnostic method to evaluate nutritional status of children. We used a newly designed transthyretin test kit to evaluate nutritional status of children admitted to our hospital. There is no national reference standard; hence we made a comparative study using anthropometric measurements and measurement of serum albumin level. A total of 102 children (51 controls and 51 study subjects) were included in this study. Transthyretin was found to be more sensitive to changes in acute malnutrition than albumin, and its level reflects recent dietary intake compared to overall nutritional status. The method is more sensitive and reliable for detection of acute malnutrition, along with anthropometric methods. Measurement of serum transthyretin level can be used as a valuable diagnostic method for assessment of acute malnutrition among children.

## 1. Introduction

Nutrition is a fundamental pillar of human life which provides adequate energy and nutrient to the cells for them to perform their physiological function of growth, reproduction, defense, and repairs [1, 2]. Malnutrition is a deficiency state of both macro- and micronutrients and their overconsumption, causing measurable adverse effects on human body structure and function resulting in specific physical and clinical outcomes. Moreover, in many developing countries, under- and overnutrition occur simultaneously [3]. Nutrition, infection, and the functions of the immune system are interrelated. Malnutrition can predispose an individual to infection and diseases and make recovery from disease slower. Infections and diseases can lead to malnutrition and nutritional deficiencies by increasing nutrients requirements, utilization, nutrients losses, and metabolism as the body tries to generate immune responses against the invading pathogens [4].

Millions of children living in low-income countries suffer from undernutrition and malnutrition, which continues to be a major public health problem in developing countries [5, 6]. Ethiopia is the second most populous country in Africa, at nearly 90 million. Approximately 14% are children under five years of age [7, 8]. Malnutrition is one of the leading causes of morbidity and mortality in children under five years of age in Ethiopia. The country has the second highest rate of malnutrition in Sub-Saharan Africa. Malnutrition in children is one of the most serious public health problems in Ethiopia and the highest in the world [6]. In fact, malnutrition is the underlying cause of 57% of child deaths in Ethiopia, with some of the highest rates of stunting and underweight in the world [9]. Problems responsible for child undernutrition include widespread poverty, poor infrastructure, high population pressure, slow economic growth, and lack of education. Others are underlying causes such as inadequate access to clean water, sanitation, food insecurity, and lack of maternal and child care services. The third groups include the highly specific risk factors like frequent infections and inadequate dietary intake [3]. The most important forms of malnutrition in Ethiopia are protein energy malnutrition (PEM), vitamin A deficiency, Iodine deficiency disorders, and Iron deficiency anemia. The problem is more severe among children aged 1–3 years who suffer from Kwashiorkor and Marasmus and underweight [10]. Nutrition outcome data which are taken from the three most recent Ethiopia Demographic and Health Surveys (EDHS) show those rates have decreased quite a bit in the past decade, most notably with mortality almost halved [11]. However, Ethiopia still needs a concerted effort to accelerate reductions in undernutrition. There is no reliable laboratory based method for assessment of malnutrition to date. Currently, anthropometric methods are widely used worldwide to assess malnutrition. Moreover, there is no reference standard for Ethiopia, be it for anthropometric or laboratory based assessment of malnutrition. In an attempt to look for a combined better assessment strategy, we designed a study for comparative study of anthropometric and laboratory based (serum transthyretin and albumin measurement) methods. This will have a paramount importance to set a range of reference standard for the different classes of malnutrition and paves the way for further investigation and information.

Transthyretin (prealbumin) is a 55 kDa, 127-amino-acid, homotetrameric serum protein produced in the liver and in small amounts in choroid plexus, pancreas, and retina but these do not affect the serum concentration [12–14]. It serves as a transport protein for thyroxine and as a carrier for retinol-binding protein (RBP) [15]. The hepatic synthesis of transthyretin necessitates a high concentration of the essential amino acid tryptophan, which plays a major role in protein synthesis initiation and is eminently sensitive to recent changes in nutritional status of both protein and energy depletion [16]. Transthyretin and retinol-binding protein both manifest rapid turnover rates, and their concentrations quickly decline in response to any change of protein nutritional status and under stressful conditions. Transthyretin is considered as a good marker for prognosis of malnutrition and monitoring refeeding efficacy and its assay serves as a key step in the assessment of nutritional status [17].

## 2. Subjects and Methods

A health institution based cross-sectional study design was conducted between August and December 2014 to assess malnutrition among children less than five years of age attending Tikur Anbessa Specialized Hospital (TASH) and Yekatit 12 Specialized Hospitals. The study population consisted of 51 malnourished and 51 normal children who had not been started on any medication or treatments. Simple random sampling technique was applied in the study period and the sample size was estimated by using a single proportion formula considering 9.7% prevalence reported by the Ministry of Health of Ethiopia and total number of children in Ethiopia less than five years of age taken as 4 million. Children attending the two hospitals and who developed acute malnutrition and children who came for vaccination but without any disease condition were taken as study subjects and controls, respectively.

### 2.1. Anthropometric Measurements

Weight was taken with children dressed lightly using portable mechanical analog scale to the nearest 0.1 Kg. Height was taken using measuring board for children less than two years of age. For children above two years of age height was taken in standing position using standard method of measurement to the nearest 0.1 cm. Data was collected by well trained nurses. Data collection form was designed to record sex, age, weight, height, arm circumference, and medical history of each child. Portable mechanical analog scales were used to measure height and weight, respectively. Using the anthropometric measures, children were classified as underweight, stunted, or wasted using the WHO international growth standards as reference.

### 2.2. Transthyretin Assay Method

Blood sample (10 *μ*l) was taken by trained nurses from finger tips of children and added to 5 ml of diluents (provided by manufacturer) with ten times gentle inversion to mix the sample properly. Then 100 *μ*l of buffer (provided by manufacturer) and 10 *μ*l of diluted sample were added to reaction well. The mixture was stirred gently with the pipette and the strip was put in the reaction well for 15 minutes. Finally after 15 minutes the strip was put to the transthyretin reading apparatus and the reading was recorded. The method utilizes immune-chromatographic assay technique using small sized test strips and a simple handset sized reading machine supplied by NIPRO Corporation. As per the manufacturers guide, cut-off point value of <100 mg/L for transthyretin indicated severe risk of protein energy malnutrition, 100–170 mg/L indicated moderate risk of protein energy malnutrition, and >170 mg/L was considered as normal.

### 2.3. Albumin Assay Method

Venous blood (3 ml) was collected from children. After 2 hours of collection of sample serum was separated and stored at −20°C in a tightly stoppered container. Albumin level was estimated using the Bromocresol green method [18]. The color of the indicator changes from yellow to blue-green when the dye binds to albumin. Working dye solution (4.0 ml) was pipetted into test tubes; then 20 *μ*l of standard, control, and test sample was added separately for each measurement and mixed properly and absorbance was measured immediately within 30 seconds at 632 nm using 6705 UV-VIS spectrophotometer (JENAWAY).

### 2.4. Ethical Consideration

Ethical clearance was obtained from the Research and Ethical Committee of the College of Health Sciences. The consent of each child's parent was obtained before inclusion of children in the study with a clear explanation of the purpose of the study to the same. Samples were collected by trained personnel observing all ethical steps and procedures.

### 2.5. Data Analysis

Collected data was coded, entered to the computer, rechecked for completeness, and analyzed using EPI-INFO and SPSS version 21. Data were expressed as mean ± standard error of mean. Differences in the means between study and control groups were evaluated by unpaired student *t*-test and chi-square test. Correlations between groups were analyzed using Pearson's correlation analysis. Graph Pad Prism version 5 was used for statistical analyses and graph drawing.

## 3. Results

The age group of children in this study ranged between one month and five years. There was a good matching with regard to age, gender, and height between the control and study groups. The overall demographic and measured data of the two groups are shown in Table 1. There were significant differences between the mean mid upper arm circumference (MUAC) value in the control group (14.32 ± 0.20 cm) and malnourished group (10.45 ± 0.20 cm) with *p* value of <0.0001. Serum albumin level measured for controls and study subjects showed that there was a significant difference between the two groups. The study group had lower mean average albumin level (3.86 ± 0.07 g/dl) than the control group (4.24 ± 0.053 g/dl) with a *p* value < 0.0001. Serum transthyretin level was also measured using a newly designed transthyretin test kit. The study group's mean value of serum transthyretin level was 132.89 ± 12.3 mg/dl and the control mean value was 303.08 ± 10.01 mg/dl with a *p* value < 0.0001, showing that the transthyretin level had really gone down for the malnourished children compared to control group.

Anthropometric and laboratory measurements were also performed to see the prevalence of malnutrition between control groups and study subjects as shown in Table 2. All children were either severely or moderately malnourished. MUAC findings showed that 39 (38.2%) children were severely malnourished and 12 (11.8%) children were moderately malnourished. All controls were normal and did not fall into any category of malnutrition. Regarding wasting (weight for height) among malnourished children 40 (78.5%) were severely malnourished and 11 (21.5%) were moderately malnourished but the controls were all in the normal range. The prevalence of underweight (weight for age) was 43 (84.3%) in severely malnourished cases and 5 (9.9%) in moderately malnourished cases and 3 (5.8%) fall under normal range. The prevalence of stunting (height for age) was 30 (58.84%) in severe malnourished cases and 15 (29.5%) in moderately malnourished cases and 6 (11.7%) fall under normal range as shown in Table 2. All of the anthropometric measurements were statistically significant (*p* < 0.001) as compared to controls. The results for anthropometric measurements showed that all controls were in the normal range but the study groups were either severely or moderately malnourished.

Transthyretin and albumin were used as biochemical markers of malnutrition to evaluate the prevalence and to categorize control group and study group into moderate, severe, and normal. Based on the cut-off point for transthyretin among the malnourished children, 7 (13.7%) were severely malnourished and 44 (86.3%) were moderately malnourished. In addition 2 (3.9%) of control group were categorized as moderately malnourished.

Using serum albumin to assess the prevalence of malnutrition with cut-off point of 3.8–5.0 g/dl as normal and <3.8 g/dl for malnutrition, 32 (62.7%) of the malnourished children were categorized as normal. This shows that albumin is a poor indicator of acute malnutrition.

Evaluation of nutritional status in different category by biochemical measurement and anthropometric measurement is illustrated in percentage in Table 3. It appeared that anthropometric measurements classified most of the study subjects as severely malnourished. Based on anthropometric measurement 84.3%, 78.4%, 76.5%, and 58.9% were classified as severely malnourished by weight for age, weight for height, MUAC, and height for age, respectively. According to our findings a better indicator for severe malnutrition seems to be the weight for age relation compared to MUAC. About 29.4%, 23.5%, 21.6%, and 9.8% were identified as moderately malnourished by height for age, MUAC, weight for height, and weight for age, respectively. In addition about 11.7% and 5.9% were classified as normal by height for age and weight for age, respectively. Through transthyretin measurement it was found that 82.4% were moderately malnourished, 13.7% were severely malnourished, and 3.9% were normal.

Categorization by albumin measurement indicated that 62.7% were normal and 37.3% were categorized as severely malnourished. From these results, it can be observed that serum transthyretin level better identifies moderately malnourished children than albumin, but albumin can identify the severely malnourished kids better than transthyretin.

Pearson correlation analyses for different anthropometric and biochemical measurements for the study and control groups were done and are shown in Table 4. Transthyretin positively correlated with nutritional indicators of serum albumin (*r* = 0.307, *p* = 0.03) in the study group. However, there is no positive correlation for control group. Additionally, we observed a negative correlation with height and weight for both control and study group, but with no statistical significance. Albumin levels correlated positively with height and weight and negatively with age for the study group, but the reverse was true for the control group.

MUAC also had a significant positive correlation with age (*r* = 0.326, *p* = 0.019), height (*r* = 0.405, *p* = 0.003), and weight (*r* = 0.430, *p* = 0.002), respectively, in the study group. Additionally, it was observed that MUAC value of control group positively correlated with transthyretin and albumin but with no statistical significance. There was no correlation between biochemical measurement and anthropometric measurements with *p* value of >0.05 for both control group and malnourished children.

## 4. Discussion

Malnutrition is a serious problem which results from a long chain of interrelated events and it continues to be a major health burden and affects mostly infants and young children in most developing countries including Ethiopia. At different occasions, many nutritional screening tools have been developed, tested, and implemented in clinical practice with several combining laboratory tests and patient information. Anthropometric measurements are widely and acceptably utilized in the assessment of malnutrition. However, it is wise and reasonable to use a combination of anthropometric and biochemical methods to reliably confirm and assess the extent of malnutrition in children. When children are admitted to hospitals, especially in developing countries, they present with acute malnutrition which complicates and tampers treatment strategies. To this effect, we made a comparative study between anthropometric and laboratory based methods to assess acute malnutrition among hospitalized children. Our results confirmed that serum transthyretin level measurement is a reliable, sensitive, and promising biochemical method useful to diagnose acute malnutrition. The test can be used for prognosis of children who are critically ill, hospitalized, and/or at risk of poor outcomes and can prompt nutritional and other support that may improve patient outcomes. A simple and handy immune-chromatographic method has been developed by a Japan based company (NIPRO corporation), which makes use of a test strip and reader with the size of a mobile phone apparatus. The test can be done in the field, laboratory, or anywhere and is very simple to operate. It requires small amount of blood taken from the fingertip (10 *μ*l) and hence it is noninvasive. The test is done on the spot and the results are also obtained in less than half an hour. Our data shows that serum transthyretin level gives better information than any other anthropometric or serum albumin estimation methods. To date there has been no reliable biochemical method for assessment of malnutrition and the anthropometric methods are also not standardized to fit to the Ethiopian situation. In addition, anthropometric determinations are prone to personal measurement errors. Therefore, serum transthyretin can be used as a good and dependable marker to assess acute malnutrition among children admitted to hospitals. The difference in physical appearance between study group and control group, identified by MUAC and transthyretin measurements, suggests that it is possible to identify malnutrition in children easily without invasive procedures.

The liver is the main site of synthesis for most plasma proteins such that low concentration of transthyretin and albumin in our study readily reflects impairment in the hepatic synthesis of plasma proteins. In malnourished children this decrease in protein synthesis is due to the limiting intake of protein. However, plasma concentrations of proteins are dependent not only on rates of synthesis but also on utilization, intravascular-extravascular transfer, catabolism, excretion, and hydration. Consequently, the overall balance between these physiological processes determines whether plasma concentration of a specific protein reflects the overall decrease in protein turnover associated with protein calorie malnutrition [19]. As the sole plasma protein expressed from birth to old age, transthyretin may be regarded as a biomarker of lean body mass and catabolic states, and it also can be used as an indicator of energy and protein adequacy in preterm, normal, and sick neonates [20, 21]. Transthyretin levels go up with increases in protein and calorie intake and decrease when protein intake is inadequate as indicated by some studies [22–25]. In one study it was shown that iron supplementation significantly increased plasma retinol, RBP, and transthyretin [26]. In this study, there were also significant differences between the mean transthyretin values in the control group and study group. The mean value of transthyretin in malnourished children was less than the average value in normal children, which is in agreement with the premises given above. Thus transthyretin is by far a better marker than albumin for severe and acute malnutrition. Perhaps it is only this protein which is a good candidate to be used as a plausible laboratory test for detection of acute malnutrition. It can also classify the different stages of malnutrition among children even better than the anthropometric methods.

## 5. Conclusion

To date the widely accepted and routinely used methods to assess malnutrition are anthropometric methods like MUAC. There is no other accepted laboratory based assay method to assess malnutrition. Transthyretin assay method is a relatively cheap, easy, and sensitive method that can be used to assess malnutrition. The very sensitive response displayed by TTR may be attributed to its small pool size, its short T_1/2_, and its high content of tryptophan. Using a biochemical marker such as transthyretin for the diagnosis of malnutrition allows many children to be evaluated for the risk of acute malnutrition regardless of underlying diagnosis of nutritional status. Therefore, the implementation of serum transthyretin level measurement in conjunction with anthropometric measurement for diagnosis of malnutrition in Ethiopian hospitals is highly recommended. Transthyretin has the potential of early identification of patients at risk of malnutrition that elude detection and provide a good picture regarding the assessment of acute malnutrition.

## Figures and Tables

**Table 1:** Comparison of mean values of anthropometric and biochemical measurement between control and study groups.

Parameter	Control (*n* = 51) Mean ± SE	Malnourished (*n* = 51) Mean ± SE	*p* value
Age (year)	1.48 ± 0.18	1.145 ± 0.13	0.13
Height (cm)	68.55 ± 2.93	61.55 ± 16.09	0.061
Weight (kg)	9.26 ± 0.59	4.5 ± 0.34	<0.0001
MUAC (cm)	14.32 ± 0.20	10.45 ± 0.20	<0.0001
Albumin (g/dl)	4.24 ± 0.053	3.86 ± 0.07	<0.0001
Transthyretin (mg/dl)	303.08 ± 10.01	132.89 ± 12.3	<0.0001

**Table 2:** Categorization of malnutrition between study and control groups using anthropometric and biochemical markers.

Variable	Total (102) *N* (%)	Control (51) *N* (%)	Malnourished (51) *N* (%)	*p* value
Gender				
Male	63 (61.8)	32 (62.7)	31 (60.7)	1.0
Female	39 (38.2)	19 (37.3)	20 (39.3)	
Height for age				
Severely malnourished	30 (29.4)	0 (0)	30 (58.8)	<0.0001
Moderately malnourished	15 (14.7)	0 (0)	15 (29.5)	
Normal	57 (55.9)	51 (89.5)	6 (11.7)	
Weight for height				
Severely malnourished	40 (39.2)	0 (0)	40 (78.5)	<0.0001
Moderately malnourished	11 (10.8)	0 (0)	11 (21.5)	
Normal	51 (50)	51 (100)	0 (0)	
Weight for age				
Severely malnourished	43 (42.15)	0 (0)	43 (84.3)	<0.0001
Moderately malnourished	5 (4.9)	0 (0)	5 (9.9)	
Normal	54 (52.95)	51 (94.4)	3 (5.8)	
MUAC				
Severely malnourished	39 (38.2)	0 (0)	39 (76.5)	<0.0001
Moderately malnourished	12 (11.8)	0 (0)	12 (23.5)	
Normal	51 (50)	51 (100)	0 (0)	
Transthyretin (prealbumin)				
Severely malnourished	7 (6.9)	0 (0)	7 (13.7)	<0.0001
Moderately malnourished	46 (45.1)	2 (3.9)	44 (86.3)	
Normal	49 (48.0)	49 (96.1)	0 (0)	
Albumin				
Normal	77 (75.5)	45 (88.2)	32 (62.7)	<0.0001
Malnutrition	25 (24.5)	6 (11.8)	19 (37.3)	

**Table 3:** Prevalence of malnutrition assessed by different methods among 51 malnourished children.

Variable	Normal %	Moderately malnourished %	Severely malnourished %
Height for age	11.7	29.4	58.9
Weight for height	0	21.6	78.4
Weight for age	5.9	9.8	84.3
MUAC	0	23.5	76.5
Transthyretin	3.9	82.4	13.7
Albumin	62.7	0	37.2

**Table 4:** Gender adjusted Pearson correlation coefficients between anthropometric and biochemical indices for malnourished children.

	Age	Height	Weight	TTR	Albumin	Age control	Weight control	Height control	TTR control	Albumin control
*R*	0.32^*∗*^	0.405^*∗∗*^	0.43^*∗∗*^	0.04	0.085	−0.08	−0.032	−0.105	−0.171	0.022
*p*	0.019	0.003	0.002	0.73	0.553	0.593	0.826	0.463	0.877	0.23

*R*	−0.05	−0.07	−0.079	0.048	0.307^*∗*^	0.026	−0.085	−0.013	0.133	0.183
*p*	0.73	0.59	0.632	0.737	0.03	0.854	0.554	0.958	0.351	0.200
*R*	−0.05	0.038	0.164	0.085	0.307^*∗*^	0.055	−0.051	−0.068	0.052	0.013
*p*	0.73	0.79	0.84	0.55	0.03	0.702	0.724	0.635	0.718	0.188

^*∗∗*^Correlation is significant at the 0.01 level (2-tailed). ^*∗*^Correlation is significant at the 0.05 level (2-tailed).
